# The German-American Medical Journal

**Published:** 1894-10

**Authors:** 


### BOOK NOTICES.

The German-American Medical Journal. Dr. Gustavus Blech and Dr. Francis T. B. Fest, editors: William C. Knocke, publisher and proprietor, No. 11 N. Broadway, St. Louis, Mo. Subscription, $1.00 per annum.

This is a new monthly designed to meet the wants of German-American physicians and surgeons in the United States and Canada.

For this purpose it is printed in the German language but not with the well-known German text. Roman is used instead. It is of the old or regular school of medicine.

The sixth number of the first volume is now before us, and is very creditable. It contains about twenty pages.

Oddly enough the pages are not numbered, and there are a few typographical errors which should be corrected.

We do not know enough German to be able to criticize its articles intelligently, so will refrain. We wish our new contemporary success and prosperity.

